# Negative mood is associated with sociobehavioral factors contributing to cardiovascular risk in an immigrant population

**DOI:** 10.1186/s12889-024-19402-z

**Published:** 2024-07-16

**Authors:** Brianna N. Tranby, Irene G. Sia, Matthew M. Clark, Paul J. Novotny, Abby M. Lohr, Laura Suarez Pardo, Christi A. Patten, Sheila O. Iteghete, Katherine A. Zeratsky, Thomas M. Rieck, Luz Molina, Graciela Porraz Capetillo, Yahye Ahmed, Hana Dirie, Mark L. Wieland

**Affiliations:** 1 Department of Psychiatry & Psychology, Mayo Clinic, 200 First Street SW, Rochester, MN 55905 USA; 2 Rochester Healthy Community Partnership, Rochester, MN USA; 3 Division of Public Health, Infectious Diseases, & Occupational Medicine, Mayo Clinic, Rochester, MN USA; 4 Department of Quantitative Health Sciences, Mayo Clinic, Rochester, MN USA; 5 Division of Epidemiology, Mayo Clinic, Rochester, MN USA; 6 Community Based Research Unit, Mayo Clinic, Rochester, MN USA; 7 Department of Endocrinology & Nutrition, Mayo Clinic, Rochester, MN USA; 8 Healthy Living Program, Mayo Clinic, Rochester, MN USA; 9 Department of Language Services, Mayo Clinic, Rochester, MN USA; 10 Somali American Social Service Association, Rochester, MN USA; 11 Division of Community Internal Medicine, Geriatrics, and Palliative Care, Mayo Clinic, Rochester, MN USA

**Keywords:** Health behaviors, Mood, Immigrant communities, Cardiovascular risk, Self-efficacy

## Abstract

**Background:**

After settling in the United States (US), immigrants often accumulate obesity and cardiovascular risk factors. As mood is often associated with health behaviors in the US population, mood may be an important mediating factor in immigrant populations.

**Methods:**

The *Healthy Immigrant Community* (HIC) study, set in southeast Minnesota, enrolled 475 adult participants in a weight loss intervention designed to reduce cardiovascular risk. Baseline questionnaires assessed mood, nutrition, physical activity, self-efficacy for healthy eating and physical activity, social support, and cohesion. A single-item mood rating of poor or fair was considered “negative”, while ratings of good, very good, or excellent were considered “positive”.

**Results:**

Hispanic/Latino (*n* = 268) and Somali (*n* = 181) adults enrolled in HIC completed baseline measures and were included in this analysis. Participants endorsing negative mood compared to positive mood had lower healthy eating scores (*p* = 0.02), lower physical activity levels (*p* = 0.03), lower confidence in eating a healthy diet (*p* = 0.001), and felt less of a sense of belonging to their community (*p* = 0.01). Those endorsing negative mood reported receiving less social support to eat healthy (*p* =  < 0.001) and be physically active (*p* = 0.01). They also accessed community resources for healthy eating (*p* = 0.001) and physical activity (*p* =  < 0.01) less frequently than participants endorsing positive mood.

**Conclusions:**

On self-report, negative mood was associated with less healthy nutrition, lower confidence in eating healthy, sedentary lifestyle, and perceived lack of belonging to the community. Integrating mood management and self-efficacy strategies may enhance the effectiveness of lifestyle interventions to reduce obesity and cardiovascular risk among immigrants who report negative mood.

**Trial registration:**

ClinicalTrials.gov registration: NCT05136339; April 23, 2022.

## Introduction

After resettling in the United States, immigrants often experience structural barriers to healthy nutrition and physical activity which leads to the accumulation of cardiovascular risk factors [1–3]. Health behaviors such as sedentary lifestyle and unhealthy nutritional intake, defined as foods with low nutrient quality or limited fruit and vegetable intake, contribute to increased rates of obesity, cardiovascular disease, and chronic health conditions [2, 3]. Our community-based participatory research (CBPR) team has engaged immigrant populations in southeast Minnesota in co-creating and implementing interventions designed to reduce obesity and cardiovascular risk factors over the past two decades [4].

In our prior research to design cardiovascular risk reduction interventions with immigrant populations, our team engaged Hispanic/Latino, Somali, and Sudanese immigrants to develop a home-based, high-contact intervention to improve nutrition and physical activity [5]. While the intervention was found to be feasible and moderately successful, post-intervention focus groups suggested involving friends and family to improve participation and target weight management. Overweight and obesity-related behaviors, normative beliefs, and intentions for control were often clustered by social networks [5].

Additionally, in a relatively small sample, our CBPR team previously found that negative mood was associated with reporting unhealthy nutritional and physical activity behaviors in both adolescent and adult immigrants [6]. Many other studies have also demonstrated an association between negative mood and poor health behaviors leading to cardiovascular disease and obesity [7–10]. In social cognitive theory, an individual’s perceived self-efficacy determines the goals they set, the amount of effort expended to meet these goals, and how they respond to challenges or failures over time [11, 12]. In turn, self-efficacy is concurrently affected by external sociocultural factors that may support or impede these goals and behaviors [13]. Thus, negative mood may lower one’s self-efficacy for healthy behaviors, leading to overconsumption of unhealthy foods and resulting in a more sedentary and isolated lifestyle.

The present Healthy Immigrant Community (HIC) randomized study used a social cognitive theoretical framework to adapt our earlier Healthy Immigrant Families study to develop a social-network delivered weight loss intervention among Hispanic/Latino and Somali immigrant communities in southeast Minnesota [14]. The purpose of this sub-analysis was to examine the possible association between self-reported negative mood with dietary quality, physical activity, self-efficacy for healthy behaviors, and sociobehavioral factors (e.g., perceived support from family and friends in healthy behaviors and sense of belonging to the community) at the time of enrollment.

## Methods

### Setting and participants

Setting and Participants

The HIC study (NCT0513633) was approved by our institution’s Institutional Research Review Board and participants provided written informed consent. The study is set in Rochester, Minnesota, a metropolitan area in southeastern MN (2022 population estimate: 121,878), and an estimated 13.6% of persons in the city were born outside of the US (2017–2021) [15]. Rochester Healthy Community Partnership (RHCP) started in 2004 with the goal of bringing together community-based organizations, community activists, and academic researchers to “improve the health of our community through CBPR, education, and civic engagement” [16]. RHCP has provided extensive training programs to local groups, improved community-based healthcare screening and delivery programs, and established a robust, productive, and cross-disciplinary research and implementation infrastructure to support community priorities [4].

The current cross-sectional analysis is embedded within the RHCP Healthy Immigrant Community study, which is a randomized, waitlist-controlled trial to assess the effectiveness of a social network-informed, CBPR-derived health promotion weight loss and cardiovascular risk reduction intervention with Hispanic/Latino and Somali immigrant communities [15]. Members of the immigrant communities receive training to become health promoters (HP) and deliver the intervention to their social networks. HPs also participate in ongoing training via bi-monthly meetings with experts in nutrition, exercise science, and behavioral weight management strategies. The intervention, delivered in 24 sessions over 12 months, consists of community-based mentoring, educational and motivational sessions, group activities, and the application of a community toolkit for healthy weight loss. A total of 51 HPs and 475 of their social network members have been enrolled and randomized in the HIC study, and the intervention is presently ongoing.

Eligibility for the HIC study included self-reported Hispanic/Latino or Somali ethnicity, age ≥ 18 years, willing to participate in all aspects of the study, and a member of a HP’s social network. Exclusion criteria was pregnancy at the time of enrollment or having a medical condition or disability that would prevent adopting a physically active lifestyle. Demographic and survey measures (e.g., dietary quality, physical activity, quality of life) and biometric measurements (e.g., height, weight, blood pressure, glucose, cholesterol) were obtained at baseline by trained study staff and community volunteers. Individuals completing baseline measures were eligible for inclusion in the current analysis.

### Primary outcome measures

#### Mood item

The baseline survey included a mood assessment item previously used by our research team: “How would you rate your mood over the past 7 days?” This question refers to things such as feeling sad, anxious, stressed, happy, etc.” [6, 17]. Based on community feedback during the designing of this item, cartoon emoji faces were created to accompany textual mood descriptor response options on a five-point Likert scale (poor; fair; good; very good; excellent). Emojis showing frowning (poor), neutral (good), and smiling (excellent) faces were placed over the text words. On analysis, responses of “poor” or “fair” were classified as having “negative mood” while responses of “good,” “very good,” or “excellent,” were classified as having “positive mood.”

#### Dietary quality and intake measurements

Nutritional behaviors were assessed using the Food Behavior Checklist, which is recommended for use among low-income and diverse communities [18, 19]. Questions such as “Do you eat fruits or vegetables as snacks” and “Do you drink regular soda” were rated on a 4-point Likert scale (no; yes, sometimes; yes, often; yes, everyday). On analysis, the “often” and “everyday” categories were combined, and three variables were assessed (no; sometimes; often).

Dietary quality and intake was also assessed by a 24-h dietary recall using the Automated Self-Administered 24-h Recall (ASA24) system, a National Cancer Institute web-based tool that enables multiple automated 24-h recalls [20]. The recalls were performed at the time of study enrollment using a computer under the supervision of study staff and with an interpreter, as needed. The ASA24® can produce several scores but the overall measure of diet is the Healthy Eating Index (HEI) [21]. The overall HEI score is comprised of 12 dietary components (e.g., total fruit, total vegetables, whole grains, dairy, total protein goods, empty calories) each with possible score from 0–10. The components are weighted equally, and the maximum overall HEI score is 100. Higher scores indicate dietary intakes in the recommended ranges.

#### Physical activity measurements

In the baseline survey, the short form of the International Physical Activity Questionnaire (IPAQ) was used to assess physical activity level [22]. Physical Activity is defined for respondents as “any activity that increases your heart rate and makes you breathe harder some of the time,” “Moderate” activity is defined as “an activity that takes *somewhat* more physical effort and makes you breathe a little harder than normal,” and “Vigorous” is described as taking “*much more* physical effort and makes you breathe *a lot* harder than normal.” Respondents indicated how many days and minutes per day they did both moderate and vigorous physical activities over the last 7 days, how many days and minutes per day were spent walking for at least 10 min over the last 7 days, and how many hours and minutes were spent sitting on a weekday over the last 7 days.

The IPAQ scoring criteria recommend three categories: Inactive, Minimally Active, and Health-Enhancing Physical Activity (HEPA) Active. “Minimally Active” is defined as “ ≥ 3 days of vigorous activity at least 20 min per day, or ≥ 5 days of moderate-intensity activity or walking at least 30 min per day, or ≥ 5 days of moderate or vigorous activity achieving a minimum of least 600 MET-min (multiples of the resting metabolic rate) per week.” Because the “minimally active” criteria exceed public health recommendations for physical activity, the categories “minimally active” and “HEPA Active” were combined on analysis and two variables were assessed (inactive; active).

### Theory-based measures

#### Self-efficacy

The adapted Patient Centered Assessment and Counseling for Exercise plus Nutrition (PACE +) survey[23] was completed at baseline, which included items on perceived self-efficacy and social support for healthy eating and physical activity. Confidence in eating a healthy diet was rated as a percentage interval (0% = not at all confident; 25%; 50% = somewhat confident; 75%; 100% = very confident). Confidence in participating in regular exercise or physical activity was rated with the same percentage interval.

#### Social support

Family and friend support for healthy eating and physical activity was also measured. The PACE + survey items “How often in the last 30 days has your family or friends encouraged you to do physical activity?” and “How often in the last 30 days has your family or friends done physical activity with you?” were rated on a 5-point Likert scale (never; once in a while; sometimes; often; always) [24]. On analysis, “never/once in a while” and “often/always” were combined, and three variables were assessed (seldom; sometimes; often). The items “How often in the last 30 days has your family or friends encouraged you to eat healthy foods?” and “How often in the last 30 days has your family or friends eaten healthy meals with you?” were rated and combined for analysis in the same manner.

#### Social cohesion

The “Social Cohesion” section of the baseline survey included the item “I feel a sense of belonging to my community” [24]. Responses were provided on a 7-point Likert scale (strongly disagree; disagree; slightly disagree; neither agree nor disagree; slightly agree; agree; strongly agree). On analysis, the three “disagreement” categories and three “agreement” categories were combined, and three variables were assessed (disagree; neither agree nor disagree; agree).

#### Resource access

Two items assessed the extent to which participants accessed existing community resources for physical activity and nutrition: “How often do you access community physical activity resources, places, or events to be physically active” and “How often do you access community nutrition resources to get healthy foods or to learn how to eat healthy”. Responses were given on a 5-point Likert scale (never; once in a while; sometimes; often; always). On analysis, “never/once in a while” and “often/always” were combined and three variables were assessed (seldom; sometimes; often).

###  Data analysis

Categorical variables classified by mood were analyzed using chi-square tests. Kruskal Wallis tests were used to analyze continuous variables by mood. Analyses were two-sided using 5% type I error rates and performed using SAS version 15.1 (SAS Institute Inc. Cary, NC, USA). No adjustments were needed due to the analysis containing only baseline data.

## Results

Of the 475 enrolled study participants, 449 completed baseline measures and were included in this analysis. Of the 449 participants, 268 (60%) were Hispanic/Latino and 181 (40%) were Somali. Although missing in 88 (19%) respondents, only 38 (11%) reported that English is the language they most commonly speak at home (11% Hispanic/Latino, 10% Somali).

Of the 449 participants, 107 (24%) reported having negative mood within the past 7 days at baseline. Participants who reported having a negative mood were more often Hispanic/Latino (74%), female (68%), had no health insurance plan in the last 12 months (46%), or were younger age (mean = 41.3 years, IQR = 32–50 years; Table 1).

**Table 1:** Healthy immigrant community participant characteristics by mood at baseline

	Negative Mood (*N* = 107)	Positive Mood (*N* = 342)	Total (*N* = 449)	*p* value
**Age**				**0.002** ^¥^
Mean (SD)	41.3 (13.2)	45.8 (14.5)	44.8 (14.3)	
Range	(18.0–79.0)	(18.0–87.0)	(18.0–87.0)	
**Gender**				0.03^*^
Missing	0	5	5	
Male	29 (27.1%)	139 (41.2%)	168 (37.8%)	
Female	73 (68.2%)	188 (55.8%)	261 (58.8%)	
Other	5 (4.7%)	10 (3.0%)	15 (3.4%)	
**Ethnicity**				0.001^*^
Hispanic/Latino	79 (73.8%)	189 (55.3%)	268 (59.7%)	
Somali	28 (26.2%)	153 (44.7%)	181 (40.3%)	
**BMI**				0.89^¥^
Mean (SD)	32.0 (6.1)	32.3 (6.7)	32.2 (6.5)	
Range	(19.2–51.9)	(16.8–80.6)	(16.8–80.6)	
**BMI Group**				0.86^*^
Missing	0	1	1	
Underweight: BMI 0 to 18.4	0 (0.0%)	2 (0.6%)	2 (0.4%)	
Heathy Weight: BMI 18.5 to 24.9	12 (11.2%)	34 (10.0%)	46 (10.3%)	
Overweight: BMI 25 to 29.9	31 (29.0%)	101 (29.6%)	132 (29.5%)	
Obese: BMI > = 30	64 (59.8%)	204 (59.8%)	268 (59.8%)	
**Have you had a health insurance plan in the past 12 months**				0.05^*^
Missing	0	5	5	
Yes	58 (54.2%)	219 (65.0%)	277 (62.4%)	
No	49 (45.8%)	118 (35.0%)	167 (37.6%)	
**How much schooling have you had**				0.26^*^
Missing	1	1	2	
High school or less	40 (37.7%)	143 (41.9%)	183 (40.9%)	
High school graduate or GED	28 (26.4%)	92 (27.0%)	120 (26.8%)	
Some college or technical degree	18 (17.0%)	67 (19.6%)	85 (19.0%)	
College or graduate school	20 (18.9%)	39 (11.4%)	59 (13.2%)	
**What is your average yearly family income**				0.49^*^
Missing	11	27	38	
$0 to $9,999	23 (24.0%)	70 (22.2%)	93 (22.6%)	
$10,000 to $19,999	8 (8.3%)	44 (14.0%)	52 (12.7%)	
$20,000 to $29,999	22 (22.9%)	61 (19.4%)	83 (20.2%)	
$30,000 to $39,999	19 (19.8%)	46 (14.6%)	65 (15.8%)	
$40,000 to $49,999	10 (10.4%)	35 (11.1%)	45 (10.9%)	
$50,000 or higher	14 (14.6%)	59 (18.7%)	73 (17.8%)	
**Which country were you born in**				0.32^*^
Missing	29	114	143	
USA	15 (19.2%)	33 (14.5%)	48 (15.7%)	
Other country	63 (80.8%)	195 (85.5%)	258 (84.3%)	
**What language do you most commonly speak at home**				0.01^*^
Missing	25	63	88	
English	10 (12.2%)	28 (10.0%)	38 (10.5%)	
Somali	15 (18.3%)	105 (37.6%)	120 (33.2%)	
Spanish	55 (67.1%)	143 (51.3%)	198 (54.8%)	
Other	2 (2.4%)	3 (1.1%)	5 (1.4%)	
**How well do you speak English**				0.58^*^
Missing	3	4	7	
Not at all	23 (22.1%)	69 (20.4%)	92 (20.8%)	
Not very well	35 (33.7%)	122 (36.1%)	157 (35.5%)	
Well	19 (18.3%)	77 (22.8%)	96 (21.7%)	
Very well	27 (26.0%)	70 (20.7%)	97 (21.9%)	
**Have you smoked at least 100 cigarettes in your entire life**				0.04^*^
Missing	5	8	13	
Yes	25 (24.5%)	52 (15.6%)	77 (17.7%)	
No	77 (75.5%)	282 (84.4%)	359 (82.3%)	
**How would you rate your physical well-being over the last seven days**				< 0.001^*^
Poor	25 (23.4%)	8 (2.3%)	33 (7.3%)	
Fair	45 (42.1%)	58 (17.0%)	103 (22.9%)	
Good	32 (29.9%)	130 (38.0%)	162 (36.1%)	
Very Good	4 (3.7%)	74 (21.6%)	78 (17.4%)	
Excellent	1 (0.9%)	72 (21.1%)	73 (16.3%)	

^¥^Kruskal Wallis

^*^Chi-Square

### Healthy dietary quality and intake

Table 2. shows the dietary results. Overall, participants who reported “no” or “sometimes” eating fruits or vegetables as snacks (*p* =  < 0.001) and “often” drinking regular soda (*p* = 0.03) were more likely to report having negative mood. Responses of “no” or “sometimes” to the item “Do you eat fruits or vegetables as snacks” were significantly associated with negative mood among both Hispanic/Latino (*p* =  < 0.01) and Somali (*p* = 0.04) participants. A lower ASA24® HEI total score, indicating less healthy nutritional intake, was also associated with negative mood for the sample overall (mean = 49.8 [IQR = 39.4–59] for negative mood vs. mean = 53.6 [IQR = 43–63.1] for positive mood; *p* = 0.02).

**Table 2.** Healthy nutrition and physical activity behaviors associated with mood at baseline

	Negative Mood (*N* = 107)	Positive Mood (*N* = 342)	Total (*N* = 449)	*p* value
**Do you eat fruits or vegetables as snacks**				** < ** 0.001^*^
Missing	1	7	8	
No	16 (15.1%)	17 (5.1%)	33 (7.5%)	
Sometimes	67 (63.2%)	193 (57.6%)	260 (59.0%)	
Often	23 (21.7%)	125 (37.3%)	148 (33.6%)	
**Do you drink fruit drinks, punch, or sports drinks**				0.09^*^
Missing	3	3	6	
No	34 (32.7%)	132 (38.9%)	166 (37.5%)	
Sometimes	52 (50.0%)	174 (51.3%)	226 (51.0%)	
Often	18 (17.3%)	33 (9.7%)	51 (11.5%)	
**Do you drink regular soda**				0.03^*^
Missing	3	0	3	
No	34 (32.7%)	139 (40.6%)	173 (38.8%)	
Sometimes	46 (44.2%)	159 (46.5%)	205 (46.0%)	
Often	24 (23.1%)	44 (12.9%)	68 (15.2%)	
**How often in the last 30 days has your family or friends encouraged you to eat healthy food**				** < ** 0.001^*^
Missing	1	2	3	
Seldom	40 (37.7%)	69 (20.3%)	109 (24.4%)	
Sometimes	30 (28.3%)	85 (25.0%)	115 (25.8%)	
Often	36 (34.0%)	186 (54.7%)	222 (49.8%)	
**How often in the last 30 days has your family or friends eaten healthy meals with you**				** < ** 0.001^*^
Missing	0	1	1	
Seldom	36 (33.6%)	70 (20.5%)	106 (23.7%)	
Sometimes	45 (42.1%)	102 (29.9%)	147 (32.8%)	
Often	26 (24.3%)	169 (49.6%)	195 (43.5%)	
**ASA24® HEI2010 TOTAL SCORE**				**0.02** ^¥^
Mean (SD)	49.8 (14.4)	53.6 (14.2)	52.7 (14.3)	
Range	(20.0–90.2)	(22.7–89.6)	(20.0–90.2)	
**How often in the last 30 days has your family or friends encouraged you to do physical activity?**				0.01*
Seldom	35 (32.7%)	72 (21.1%)	107 (23.8%)	
Sometimes	34 (31.8%)	98 (28.7%)	132 (29.4%)	
Often	38 (35.5%)	172 (50.3%)	210 (46.8%)	
**How often in the last 30 days has your family or friends done physical activity with you?**				** < ** 0.001*
Seldom	64 (59.8%)	127 (37.1%)	191 (42.5%)	
Sometimes	22 (20.6%)	108 (31.6%)	130 (29.0%)	
Often	21 (19.6%)	107 (31.3%)	128 (28.5%)	
**IPAQ Physical Activity Category**				0.03*
Missing	5	14	19	
IPAQ: Inactive	40 (39.2%)	91 (27.7%)	131 (30.5%)	
IPAQ: Minimally Active or HEPA Active	62 (60.8%)	237 (72.3%)	299 (69.5%)	

^*^Chi-Square

^¥^Kruskal Wallis

### Physical activity

Participants who were classified in the “inactive” category more often reported negative mood compared to positive mood (39% vs 28%), while participants who were classified in the “minimally or HEPA active” category more often reported positive mood compared to negative mood (72% vs 61%; *p* = 0.03). Somali participants who were classified in the “inactive” category more often reported negative mood (62% vs 28%), while those in the “minimally or HEPA active” category more often reported positive mood (72% vs 39%; *p* = 0.001), but there was no significant difference among Hispanic/Latino participants.

### Theory-based measures

Table 3. shows results from the social cognitive theory-based measures of perceived self-efficacy, social support, and social cohesion.

**Table 3.** Self-efficacy, social support, social cohesion, and resource access associated with mood at baseline

	Negative Mood (*N* = 107)	Positive Mood (*N* = 342)	Total (*N* = 449)	*p* value
Self-Efficacy				
**How confident are you that you can eat a healthy diet**				0.001^*^
Missing	0	1	1	
0% Not at All Confident	2 (1.9%)	4 (1.2%)	6 (1.3%)	
25%	9 (8.4%)	17 (5.0%)	26 (5.8%)	
50% Somewhat Confident	47 (43.9%)	84 (24.6%)	131 (29.2%)	
75%	21 (19.6%)	97 (28.4%)	118 (26.3%)	
100% Very Confident	28 (26.2%)	139 (40.8%)	167 (37.3%)	
**How confident are you that you can participate in regular exercise or physical activity**				0.09^*^
Missing	0	1	1	
0% Not at All Confident	5 (4.7%)	12 (3.5%)	17 (3.8%)	
25%	10 (9.3%)	24 (7.0%)	34 (7.6%)	
50% Somewhat Confident	41 (38.3%)	91 (26.7%)	132 (29.5%)	
75%	24 (22.4%)	91 (26.7%)	115 (25.7%)	
100% Very Confident	27 (25.2%)	123 (36.1%)	150 (33.5%)	
Social Support				
**How often in the last 30 days has your family or friends encouraged you to eat healthy food**				** < ** 0.001^*^
Missing	1	2	3	
Seldom	40 (37.7%)	69 (20.3%)	109 (24.4%)	
Sometimes	30 (28.3%)	85 (25.0%)	115 (25.8%)	
Often	36 (34.0%)	186 (54.7%)	222 (49.8%)	
**How often in the last 30 days has your family or friends eaten healthy meals with you**				** < ** 0.001^*^
Missing	0	1	1	
Seldom	36 (33.6%)	70 (20.5%)	106 (23.7%)	
Sometimes	45 (42.1%)	102 (29.9%)	147 (32.8%)	
Often	26 (24.3%)	169 (49.6%)	195 (43.5%)	
**How often in the last 30 days has your family or friends encouraged you to do physical activity**				0.01^*^
Seldom	35 (32.7%)	72 (21.1%)	107 (23.8%)	
Sometimes	34 (31.8%)	98 (28.7%)	132 (29.4%)	
Often	38 (35.5%)	172 (50.3%)	210 (46.8%)	
**How often in the last 30 days has your family or friends done physical activity with you**				** < ** 0.001^*^
Seldom	64 (59.8%)	127 (37.1%)	191 (42.5%)	
Sometimes	22 (20.6%)	108 (31.6%)	130 (29.0%)	
Often	21 (19.6%)	107 (31.3%)	128 (28.5%)	
Social Cohesion				
**I feel a sense of belonging to my community**				0.01^*^
Missing	0	3	3	
Disagree	14 (13.1%)	23 (6.8%)	37 (8.3%)	
Neither Agree nor Disagree	18 (16.8%)	32 (9.4%)	50 (11.2%)	
Agree	75 (70.1%)	284 (83.8%)	359 (80.5%)	
Resource Access				
**How often do you access community physical activity resources, places, or events to be physically active**				0.003^*^
Missing	0	2	2	
Seldom	56 (52.3%)	124 (36.5%)	180 (40.3%)	
Sometimes	34 (31.8%)	110 (32.4%)	144 (32.2%)	
Often	17 (15.9%)	106 (31.2%)	123 (27.5%)	
**How often do you access community nutrition resources to get healthy foods or learn how to eat healthy**				0.001^*^
Seldom	68 (63.6%)	157 (45.9%)	225 (50.1%)	
Sometimes	26 (24.3%)	87 (25.4%)	113 (25.2%)	
Often	13 (12.1%)	98 (28.7%)	111 (24.7%)	

^*^Chi-Square

#### Self-efficacy

For the sample overall, participants who reported being not at all or somewhat confident in eating a healthy diet were more likely to report negative mood than those who reported being very confident (*p* = 0.001). Among Hispanic/Latino participants, those who reported being not at all or somewhat confident in eating a healthy diet were more likely to report negative mood (*p* =  < 0.001), but there was no significant difference among Somali participants.

#### Social support

Overall, participants who reported “seldom” or “sometimes” having family or friends encourage them to eat healthy food in the last 30 days (*p* =  < 0.001) or eat healthy meals with them in the last 30 days (*p* =  < 0.001) were more likely to report having negative mood compared to positive mood. Among Hispanic/Latino participants, those who reported “seldom” or “sometimes” having family or friends encourage them to eat healthy foods in the last 30 days (*p* = 0.01) or eating healthy meals with them in the last 30 days (*p* = 0.001) were more likely to report negative mood. No significant difference was detected among Somali participants.

Participants who reported “seldom” or “sometimes” having family or friends encourage them to do physical activity in the last 30 days were more likely to report negative mood (*p* = 0.01). Those who reported having family or friends only “seldom” actually do physical activities with them in the last 30 days also reported negative mood more often (*p* =  < 0.001) than those reporting a positive mood. Among both Hispanic/Latino and Somali participants, only the question on having family or friends do physical activity with them in the last 30 days and the response category of “seldom” was statistically significant (*p* = 0.01 and 0.05, respectively) in being associated with negative mood.

#### Social cohesion and resource access

Participants who either “disagreed” or “neither agreed nor disagreed” with the statement “I feel a sense of belonging to my community” reported negative mood more often than participants with higher social cohesion ratings (*p* = 0.01). For the sample overall, those who “seldom” or “sometimes” access community resources to get healthy foods or learn to eat healthy (*p* = 0.001) or who “seldom” or “sometimes” access community physical activity resources (*p* = 0.003) reported negative mood more than participants who “often” access community resources.

For both Hispanic/Latino and Somali participants, the question “How often do you access community nutrition resources to get healthy foods or to learn how to eat healthy?” was significantly associated with negative mood. Hispanic/Latino participants who reported “seldom” accessing community nutrition resources (*p* = 0.01) and Somali participants who reported “seldom” or “sometimes” accessing community nutrition resources (*p* = 0.03) were more likely to report negative mood. Additionally, Somali participants who responded “seldom” or “sometimes” to the question “How often do you access community physical activity resources, places, or events to be physically active?” more often reported negative mood (*p* 0.02), but there was no significant difference among Hispanic/Latino participants.

## Discussion

This study assessed the baseline measures of Hispanic/Latino and Somali immigrants enrolled in a CBPR-derived weight loss intervention program designed to reduce cardiovascular risk factors. Self-reported negative mood was associated with several risk factors associated with cardiovascular disease including poor dietary quality, low physical activity levels, and a lack of perceived social support. These findings confirm results from a prior smaller study by our research team among adolescents and adults from shared households, and extend the findings to a larger adult immigrant population [6]. Yet, further research is needed in this area as a literature search yielded only two studies published since our 2017 report which identified mood as a facilitator of healthy lifestyle behavior among immigrant populations. One study examined nutrition and one examined physical activity; no studies on social support facilitating health behaviors among immigrant populations were noted [25, 26].

It is well-established that self-efficacy, or one’s perceived ability to exert control over their behavior, mood, and perceived social support, are associated with future health behaviors [27–29]. In examining dietary quality, study participants classified as having negative mood reported eating fewer fruits and vegetables, drinking more regular soda, and had less healthy diets as measured by the 24-h dietary recall (ASA24). They also reported lower confidence in eating healthfully compared to participants reporting a positive mood. Additionally, participants reporting a negative mood endorsed receiving less support for healthy eating (e.g., encouragement from family or friends to eat healthy food, frequency of family or friends eating healthy meals with them) compared to those reporting a positive mood. Given the significant relationship between self-efficacy and health behaviors, individuals reporting negative mood are likely to continue to have poor dietary quality and low physical activity levels in the future [29, 30].

Similar results were found in the domain of physical activity. Study participants classified as having a negative mood were more likely to be physically inactive compared to those with a positive mood. Previous research has found that physical activity can benefit those with negative moods, and may offer protection from the development of depression [31, 32]. Participants in our study endorsing negative mood also reported receiving less encouragement to be physically active from family or friends, and having others do physical activities with them. This aligns with previous research that has found that friends and family who are obese may influence the obesity rates of those around them [33], and conversely, that membership in a sport or exercise group can be beneficial to both mood and physical activity [34]. Thus, interventions that seek to improve physical activity levels in immigrants reporting negative mood should address social support for, and self-confidence in, being physically active.

Social support and social cohesion are associated with a range of positive health behaviors and outcomes [35]. In this study, participants classified as having negative mood also reported a lower sense of community belonging compared to participants with a positive mood. Those with negative mood were also less likely to report accessing community resources for healthy eating and physical activity. The finding that a lack of community belongingness is associated with a lower level of accessing community resources is in line with other research supporting the strong association between social support and positive health behaviors [36].

The results presented herein confirm prior findings supporting an association between mood, healthy diet, and physical activity, and reflect the self-reported experiences of Hispanic/Latino and Somali immigrants prior to starting a healthy lifestyle intervention. The study has several limitations. The results are limited by measures of some health behaviors being self-reported, as it is possible that direct measurement of dietary quality and intake or physical activity level would yield different findings. Additionally, as the measure for mood classification was a single item, it is possible that a more extensive assessment of mood may have classified participants differently. Furthermore, the US version of the ASA24 is available in Spanish, but Somali participants had some difficulty reporting food intake due to cultural differences. Participants were individuals in southeast Minnesota who self-enrolled in a weight loss intervention and may differ from the broader immigrant population. It is possible that study participants had more access to community or healthcare resources. This potential difference warrants investigation. Future researchers should consider incorporating direct measures of diet and physical activity, and the use of structured, clinical diagnostic interviews or clinical questionnaires to assess mood. Participants in this study have experienced heterogeneous cultural, linguistic, and migration-related contexts. However, they share a collective migration experience to the United States, where immigration status can be conceived as a social determinant of health [37]. Immigrants, as historically marginalized populations, face structural barriers to health promoting environments and programs. Data from this study provide specific strategies to build into future health promotion programs with immigrant communities, regardless of cultural context; namely, integration of mood management and self-efficacy strategies. These approaches should be assessed longitudinally in future studies to model additional mechanistic evidence.

In conclusion, in this baseline assessment in Hispanic/Latino and Somali immigrants, self-reporting a negative mood was associated with a less healthy diet, lower physical activity levels, and lower levels of confidence in eating a healthy diet and being physically active. Additionally, participants reporting negative mood had less of a sense of belonging to their communities and were less likely to access community resources for healthy eating or physical activity. While more research is certainly needed in larger and other immigrant communities, our findings suggest that health-improvement behavioral interventions may benefit from incorporating mood management and self-efficacy strategies as additional mechanisms to reduce cardiovascular risk factors and promote health equity.

## Data Availability

The data that support the findings of this study are available from the corresponding author, BNT, upon reasonable request.
